# Transcriptomic Biomarkers for Tuberculosis: Evaluation of *DOCK9. EPHA4*, and *NPC2* mRNA Expression in Peripheral Blood

**DOI:** 10.3389/fmicb.2016.01586

**Published:** 2016-10-25

**Authors:** Leonardo S. de Araujo, Lea A. I. Vaas, Marcelo Ribeiro-Alves, Robert Geffers, Fernanda C. Q. Mello, Alexandre S. de Almeida, Adriana da S. R. Moreira, Afrânio L. Kritski, José R. Lapa e Silva, Milton O. Moraes, Frank Pessler, Maria H. F. Saad

**Affiliations:** ^1^Laboratório de Microbiologia Celular, Fundação Oswaldo Cruz, Instituto Oswaldo CruzRio de Janeiro, Brazil; ^2^TWINCORE, Center for Experimental and Clinical Infection ResearchHannover, Germany; ^3^Laboratório de Pesquisa Clínica em DST-AIDS, Fundação Oswaldo Cruz, Instituto de Pesquisa Clínica Evandro ChagasRio de Janeiro, Brazil; ^4^Helmholtz Centre for Infection ResearchBraunschweig, Germany; ^5^Thoracic Diseases Institute, Federal University of Rio de JaneiroRio de Janeiro, Brazil; ^6^Laboratório de Hanseníase, Fundação Oswaldo Cruz, Instituto Oswaldo CruzRio de Janeiro, Brazil

**Keywords:** *M. tuberculosis*, LTBI, tuberculosis, messenger RNA, biomarkers

## Abstract

Lately, much effort has been made to find mRNA biomarkers for tuberculosis (TB) disease/infection with microarray-based approaches. In a pilot investigation, through RNA sequencing technology, we observed a prominent modulation of *DOCK9, EPHA4*, and *NPC2* mRNA abundance in the blood of TB patients. To corroborate these findings, independent validations were performed in cohorts from different areas. Gene expression levels in blood were evaluated by quantitative real-time PCR (Brazil, *n* = 129) or reanalysis of public microarray data (UK: *n* = 96; South Africa: *n* = 51; Germany: *n* = 26; and UK/France: *n* = 63). In the Brazilian cohort, significant modulation of all target-genes was observed comparing TB vs. healthy recent close TB contacts (rCt). With a 92% specificity, *NPC2* mRNA high expression (*NPC2*^high^) showed the highest sensitivity (85%, 95% CI 65%–96%; area under the ROC curve [AUROC] = 0.88), followed by *EPHA4* (53%, 95% CI 33%–73%, AUROC = 0.73) and *DOCK9* (19%, 95% CI 7%–40%; AUROC = 0.66). All the other reanalyzed cohorts corroborated the potential of *NPC2*^high^ as a biomarker for TB (sensitivity: 82–100%; specificity: 94–97%). An *NPC2*^high^ profile was also observed in 60% (29/48) of the tuberculin skin test positive rCt, and additional follow-up evaluation revealed changes in the expression levels of *NPC2* during the different stages of *Mycobacterium tuberculosis* infection, suggesting that further studies are needed to evaluate modulation of this gene during latent TB and/or progression to active disease. Considering its high specificity, our data indicate, for the first time, that *NPC2*^high^ might serve as an accurate single-gene biomarker for TB.

## Introduction

*Mycobacterium tuberculosis* bacilli spread through the air, and the lung parenchyma is the main site of infection. Despite the availability of effective treatment, new tuberculosis (TB) cases are commonly mis- or under-diagnosed, leading to delayed initiation of chemotherapy and, consequently, to high morbidity and mortality (Golub et al., 2005; Belay et al., 2012). The diagnosis of pulmonary TB relies on the combination of clinical and epidemiological features associated with chest radiographic changes and microbiological findings (detection of acid-fast bacilli, culture growth or nucleic acid detection; Sia and Wieland, 2011). Nevertheless, the sputum smear lacks in sensitivity (50–70%), and the more sensitive culture (80–89% sensitivity), the gold-standard method, is time consuming as 4–8 weeks is required for visible colonies to appear (Reichman and Hersfield, 1993; Sia and Wieland, 2011). The availability of commercial automated rapid molecular tests (Xpert^®^ MTB/RIF) has overcome this problem, providing results within 2 h and showing moderate to high accuracy (61.1–100% sensitivity; 90.9–100% specificity; Rachow et al., 2011). However, the use of any of these tests to investigate suspected cases of pulmonary TB is more complicated if sputum is of low quality or quantity, routinely requiring a bronchoscopy with bronchoalveolar lavage (Khalil and Butt, 2015). Thus, the global search for fast, more accurate and non-invasive diagnostic biomarkers for TB continues.

As the transcription of genes is a very dynamic process, modulation of their expression is the first major regulatory change in many biological processes (Riedmaier and Pfaffl, 2013). Correspondingly, prominent gene expression regulation has been found in blood cells of patients with pulmonary TB or LTBI (Berry et al., 2010; Lu et al., 2011; Maertzdorf et al., 2011, 2012; Bloom et al., 2013; Anderson et al., 2014; Esterhuyse et al., 2015; Laux da Costa et al., 2015; Zak et al., 2016). The potential applications of biomarkers for TB include not only the improvement of the current diagnostic tests, but also the possibility of assessing the response to drug therapy and targeted screening of eligible subjects for new TB vaccines and therapeutic trials. However, most previous exploratory attempts are based on array techniques for mRNA expression, which may have contributed to a high redundancy among studies, usually revealing mRNAs whose products correlate with the human immune response to TB: type-I and type-II interferon signaling, Toll-like receptors, T- and B-cell functions, and neutrophil influx (Blankley et al., 2014; Deffur et al., 2015). Moreover, even though some of the presented biomarkers/biosignatures showed high accuracy for TB detection, the lack of validations in cohorts from different geographical areas and/or the proposed use of combinations of a number of genes (from sets of 3 to 100s), hinder their use in clinical routine.

The advent of RNA sequencing (RNAseq) technology allowed the simultaneous measurement and profiling of the transcriptome of a biosample, avoiding biases introduced during microarray hybridization (Zhao et al., 2014). The aim of the present study therefore was to identify new mRNA biomarkers for TB in whole blood specimens. For this purpose, in an exploratory investigation, a small set of recent close TB contacts (rCt) exposed to a bacillary index case, and TB patients, were submitted to whole blood transcriptomic profiling via RNAseq (unpublished data); the raw transcriptome data can be found at the NCBI Gene Expression Omnibus (GEO) under the accession code GSE84076. Mining these data, highly prominent regulation of *DOCK9, EPHA4*, and *NPC2* mRNA expression was observed in the TB samples. Following a first validation by quantitative real-time PCR (RT-qPCR) in a cohort of rCt and TB cases from Rio de Janeiro/Brazil, expression data from previous microarray studies (Berry et al., 2010; Maertzdorf et al., 2012; Bloom et al., 2013) were also used for an independent validation of these potential mRNA biomarkers in populations with different levels of TB incidence and diverse genetic backgrounds.

## Materials and Methods

### Ethics Statements

The study protocol was approved under the registration codes 560-10, 183-10, and 190/12, by the Ethics Committee of the Oswaldo Cruz Foundation, Clementino Fraga Filho University Hospital (CFFUH) and Polyclinic Augusto do Amaral Peixoto (PAAP), respectively. Participation in this study was voluntary and only subjects ≥18 years of age who gave informed written consent were eligible.

### Participants

All participants were recruited from March 2010 to November 2013 at CFFUH and PAAP, both located in Rio de Janeiro state, Brazil, a TB endemic area with an incidence rate of 60.9/100,000 inhabitants (Equipe Gerência de Pneumologia Sanitária SES/RJ, 2014). Demographic characteristics were gathered via a structured questionnaire.

According to the Brazilian Ministry of Health policy recommendations for TB/LTBI screening, all rCt recruited underwent a clinical exam, the Mendel-Mantoux tuberculin skin test (TST), and chest X-ray examination at enrollment (T_0_). Besides clinical and epidemiological data, the diagnostic criteria for TB among rCt or index cases were at least one acid-fast bacilli-positive sputum sample for *M. tuberculosis* and/or positive sputum culture.

After TB was excluded, TST results were read after 72 h, and any rCt showing an induration ≥5 mm were considered as LTBI. The rCt TST negative individuals (TST^neg^) were followed up and retested after 12 months (T_12_). For those rCt showing a positive TST (TST^pos^) or conversion (increase in induration of the TST reaction of at least 10 mm) at any time-point, a free-of-cost prophylactic treatment for LTBI (tLTBI) was offered, as well as the appropriate treatment for individuals who developed TB (TB progressors).

For the pilot RNAseq investigation a group of rCt from the Brazilian cohort was stratified according to the response to both TST and in house interferon-gamma release assays (IGRAs) under ESAT6:CFP10 stimulation (Araujo et al., 2014). Control and LTBI groups comprised 12 and 16 participants with TST^neg^/IGRA^neg^ or TST^pos^/IGRA^pos^, respectively. Six confirmed TB cases were also included.

The validation by RT-qPCR was performed using a larger cohort of rCt where IGRA results were not available. As the participants presented different baseline and follow-up responses to TST, they were allocated into groups according to the probability of having LTBI: (i) G.I (*n* = 12), very low: no TST reaction (0 mm) at baseline and at any of the follow up retests; (ii) G.II (*n* = 24), low to moderate: baseline TST^neg^ (0–4 mm) who did not return for skin test re-evaluation (16/24, 67%) or converted to TST^pos^ (8/24, 33%); and (iii) G.III (*n* = 48), high: baseline TST^pos^ (TST > 5 mm). The majority of TB samples (26/45, 58%) were from treatment-naive participants, and together with those treated ≤2 days comprise the group G.IV (*n* = 29). G.V (*n* = 12) comprises samples from TB cases with 3–6 days of anti-TB treatment, and G.VI consists of samples from TB cases with 7 days (*n* = 2) or 6 months (*n* = 2) of anti-TB treatment. Characteristics of all study participants are summarized in **Table 1**.

**Table 1:** Clinical and demographic characteristics of the study population.

Groups	*n*	Age^1^ (SD)	Males (%)	TST (T_0_)^2^	TST (T_4_ or T_12_)^2^	Abnormal Chest X-ray	Symptoms of TB	Sputum-smear/(and/or) culture positive cases
**Probability of LTBI**								
G.I Very low	12	38.3 (16.2)	1 (8.3)	0	0	0	0	0/0
G.II Low/moderate	24	40.1 (15.5)	6 (25)	0–4	ND or ≥5 mm	0	2	0/0
G.III High	48	41.9 (14.9)	14 (29)	≥5	–	6	12	0/0
**TB (days after treatment onset)**								
G.IV 0–2	29	39.4 (14.9)	14 (48)	–	–	29	29	18/18
G.V 3–6	12	43.6 (13.1)	8 (67)	–	–	12	12	10/6
G.VI ≥ 7	4	47.5 (20.1)	3 (75)	–	–	2	4	4/0^3^
**Total**	**129**	41.1 (14.9)	46 (36)	–	–	49	59	–


*The recent close tuberculosis (TB) contacts (rCt) and pulmonary TB patients were stratified according to the probability of latent TB infection (LTBI), via tuberculin skin test (TST) response at enrollment (T_*0*_) and follow up (T_*4*_ or T_*1*2_), or the time (days) relapsed between the anti-tuberculosis treatment onset and the blood collection for this study, respectively.*

*^1^Mean age and standard deviation (SD), in years. ^2^TST in millimeters; Follow up TST in the 4th or 12th consecutive month. ^3^At the time of diagnostic. ND, not done.*

### Specimen Collection and RNA Isolation

Peripheral whole blood was collected in Paxgene RNA tubes (PreAnalytiX, SWZ) and stored at -70°C until processing. Total RNA was isolated using the PAXgene Blood miRNA Kit (PreAnalytiX, SWZ), which is indicated for the isolation and purification of total RNA longer than 18 nucleotides. The manufacturer’s instructions were followed at both stages. Total RNA was quantified with a Nanodrop ND-1000 spectrophotometer (Thermo Scientific, EUA) and RNA integrity was assessed via agarose gel electrophoresis.

### cDNA Synthesis and RT-qPCR

*DOCK9. EPHA4*, and *NPC2* mRNAs were selected for further validations. Total RNA (100 ng) was reverse transcribed into complementary DNA (cDNA) using the oligo (dT) primer and Superscript III kits (Invitrogen, Life Technologies, USA), according to the manufacturer’s protocol. RT-qPCR was performed as previously reported (Guerreiro et al., 2013), with minor modifications in the master mix preparation: 0.4 μL (250 nM) of gene-specific primers (**Supplementary Table S1**); 10 μL of Fast SYBR green (Applied Biosystems, Molecular Probes, Inc.); 2 μL of cDNA; and 7.6 μL of sterile DNase-, RNase-, and DNA-free water. All RT-qPCR tests were performed in duplicate, assayed and analyzed in a blind manner.

### RT-qPCR Relative Expression Analysis

Individual expression of *DOCK9. EPHA4*, and *NPC2* mRNAs was obtained as crossing point (Cp) values (Guerreiro et al., 2013), which were subsequently normalized to *RPL13A* mRNA expression, using the qPCR library (Ritz and Spiess, 2008) for the R statistical package 3.2.3 (R Development Core Team, 2014), resulting in relative expression data for the three target genes.

### Microarray Reanalysis

Whole blood normalized microarray expression data from HD, LTBI and pulmonary TB adults (≥18 years of age) available at the GEO under accession number GSE19491 (Berry et al., 2010), GSE34608 (Maertzdorf et al., 2012), and GSE42826 (Bloom et al., 2013) were exported to Prism 6 (GraphPad Software) using the GEO2R (Barrett et al., 2013) web tool. Follow up data during anti-TB treatment were also available in Berry et al. (2010).

### Statistical Analysis

Differences among biological groups were assessed with the Mann–Whitney (two groups) or Kruskal–Wallis (more than two groups) tests followed by Dunn’s multiple comparison tests correction, when applicable. The Friedman test was applied to analyze follow up data. *p*-values ≤ 0.05 and *q*-values < 0.01 were considered as significant. Mean, median, standard deviation (SD), dispersion plots, AUROC (area under the receiver operating characteristics curve) values and 95% confidence intervals (CI) were computed using Prism 6 (GraphPad Software); while the R statistical package 3.2.3 (R Development Core Team, 2014) was used for the ROC (receiver operating characteristics) curves (Robin et al., 2011), classification tree analysis (Therneau et al., 2015), and Venn diagrams (Chen and Boutros, 2011). Cut-off points were selected by fixing specificities ≥90%, after which relative expression of each gene was classified as low (below the cut-off) or high (above the cut-off).

## Results

### Target-Genes Screening via RNAseq

The proposed groups for this pilot investigation did not differ significantly in age (mean age [SD], in years: control: 42.1 [14.4]; LTBI: 45.5 [12.7]; and TB: 44.9 [13.2], *p* = 0.202). A predominance of women among control (10/12, 83%) and LTBI (10/16, 63%) groups compared to the active TB cases (2/6, 33%) was observed.

**FIGURE 1:**
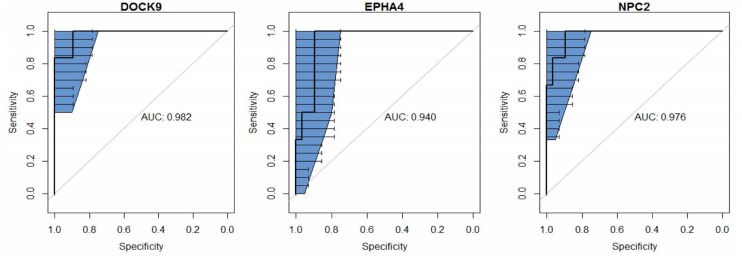
**Receiver operating characteristic (ROC) curve analysis and the respective areas under the curve (AUC) with 95% confidence intervals (blue), comparing peripheral blood *DOCK9. EPHA4*, and *NPC2* gene expression profiles, obtained via RNA sequencing, between recent close tuberculosis (TB) contacts (rCt, *n* = 28) and patients with pulmonary TB (*n* = 6) for the screening of target genes for *Mycobacterium tuberculosis* infection**.

The group with active pulmonary TB was used as reference of infection for biomarker screening. Compared to control and LTBI groups, 120 and and 52 genes, respectively, were differentially expressed in the TB cases. From these sets of genes, 13 mRNAs (*ANKRD22. APOL4. BANK1. BATF2. DHRS9. DOCK9. EPHA4. ETV7. FAM26. FMN1. NPC2. NT5E*, and *WARS*) were found in both comparisons (TB vs. LTBI or controls). Then, as the highest AUROC (≥0.94) were observed for *DOCK9. EPHA4*, and *NPC2* (**Figure 1**), these three genes were selected for further validations.

### Gene Expression Profiling via RT-qPCR: The Brazilian Cohort

The assay was performed in 129 participants comprising 45 TB patients and 84 rCt. The majority of the TB cases were confirmed by a positive sputum smear and/or culture (G.IV = 27/29 [93%]; G.V = 10/12 [83%]; G.VI = 3/4 [75%]). Clinical examination and free-of-cost treatment for LTBI/TB was offered at baseline and during follow up, but 35% (29/84) of the rCt did not return for re-evaluation. The mean age [SD] of the recruited rCt (41 [15.1] years) and TB (41 [14.9] years) participants was not significantly different (*p* = 0.787). Most of the TB patients were males (rCt: 21/84, 25%; TB: 25/45, 56%) with abnormal findings on chest x-ray (rCt: 6/84, 7%; TB: 45/45, 100%) and clinical symptoms of TB (rCt: 14/84, 17%; TB: 43/45, 96%; **Table 1**).

**FIGURE 2:**
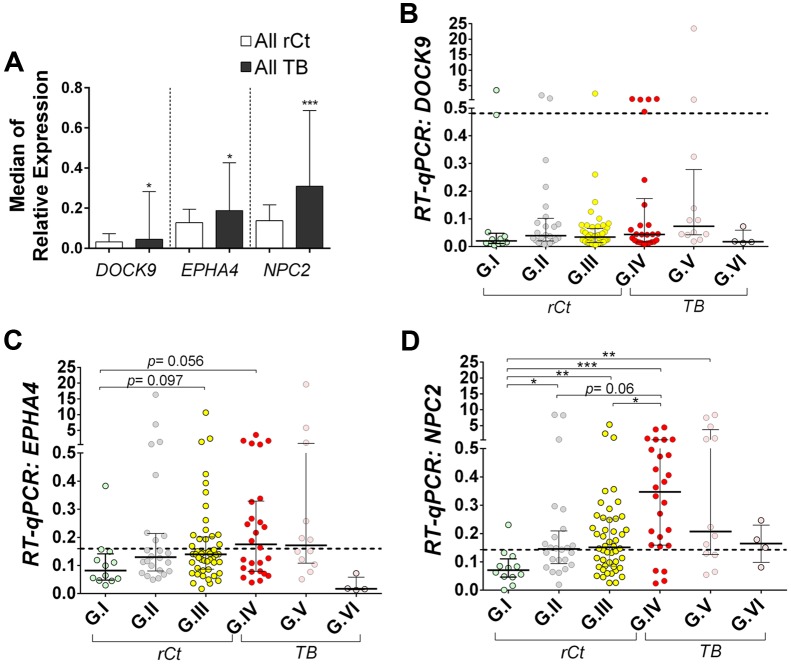
**Relative expression of *DOCK9, EPHA4*, and *NPC2* mRNAs in whole blood, as obtained with quantitative real-time PCR (RT-qPCR).** *RPL13A* mRNA was used as reference (housekeeping) gene. All participants were recruited from the broad population of routine clinical practice at public hospitals in Rio de Janeiro/Brazil. Data are shown as median ± interquartile range. **(A)** Comparison between all healthy recent close TB contacts (rCt) and patients with pulmonary tuberculosis (TB). Mann–Whitney *U*-test; ^∗^*p* < 0.05 and ^∗∗∗^*p* < 0.001. **(B–D)** Comparison between healthy recent close TB contacts (rCt) stratified in three groups according to probability of latent tuberculosis infection: (G.I, *n* = 12) very low, (G.II, *n* = 24) low to moderate, and (G.III, *n* = 48) moderate to high; and tuberculosis (TB) cases grouped by the time elapsed between the anti-TB treatment onset and the blood collection for this study (G.IV: 0–2 days, *n* = 29; G.V: 3–6 days, *n* = 12; and G.VI: ≥7 days, *n* = 4). **(B)** *DOCK9*, **(C)** *EPHA4*, and **(D)** *NPC2* gene expression. The Kruskal–Wallis test was used to access significance of global differences across groups (*DOCK9 p* = 0.039, *EPHA4 p* = 0.059 and *NPC2 p* < 0.0001), among rCt (*DOCK9 p* = 0.276, *EPHA4 p* = 0.079 and *NPC2 p* = 0.005) or TB (DOCK9: *p* = 0.18; EPHA4: *p* = 0.38; and NPC2: *p* = 0.28), followed by Dunn’s Multiple Comparison test (^∗^*p* < 0.05, ^∗∗^*p* ≤0.005, and ^∗∗∗^*p* ≤ 0.001).

Gene expression profiling of *EPHA4* and *NPC2* showed a tendency of gradual median increment from G.I to G.IV, decreasing toward G.V and G.VI (**Supplementary Table S2**). Hence, in multiple group comparisons, only *EPHA4* and *NPC2* offered significant, or borderline, differences between controls (G.I) and latently (G.III) or actively infected groups (G.IV) (**Figures 2B–D**). Interestingly, a bimodal expression profile was observed for *DOCK9* and *EPHA4* especially in group G.IV (**Figures 1** and **2B**).

*NPC2* presented the most significant differential expression, as mRNA levels in controls (G.I) were significantly lower than in the other groups (*p* < 0.05), except for TB with >7 days of specific treatment (G.VI, *p* > 0.999). Moreover, up-regulation of *NPC2*, with consistent changes in median expression, was observed in active disease (G.IV, *p* ≤ 0.001) compared to rCt with high (G.III, *p* < 0.05) or low-to-moderate (G.II, *p* = 0.06) probability of infection (**Figure 2D**).

### RT-qPCR: ROC Curve Analysis

Receiver operating characteristic curve analysis demonstrated that relative expression of *NPC2* mRNA had the highest discriminatory power for detection of LTBI (G.III, AUROC = 0.79, 95% CI = 0.65–0.92), TB (G.IV, AUROC = 0.88, 95% CI = 0.77–0.99), or both active and latent infections (G.III + G.IV, AUROC = 0.82, 95% CI = 0.72–0.93), followed by *EPHA4* (LTBI, G.III: AUROC = 0.70, 95% CI = 0.53–0.87; TB, G.IV: AUROC = 0.73, 95% CI = 0.56–0.90; and both G.III + G.IV: AUROC = 0.720, 95% CI = 0.56–0.88) and last by *DOCK9* (LTBI, G.III: AUROC = 0.59, 95% CI = 0.40–0.79; TB, G.IV: AUROC = 0.66, 95% CI = 0.46–0.86; and both G.III + G.IV: AUROC = 0.63, 95% CI = 0.43–0.82) (**Supplementary Figure S1**).

**FIGURE 3:**
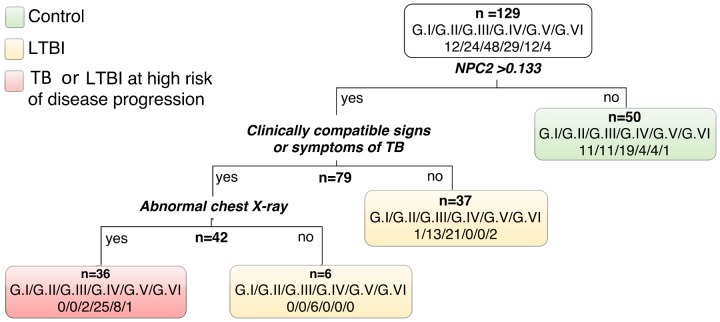
**Classification tree using relative expression of *NPC2* mRNA by RT-qPCR (cut-off 0.133) in conjunction with clinical and radiological information.** Latent TB infection probability groups: G.I very low, G.II low to moderate, and G.III moderate to high; and patients with active pulmonary tuberculosis (TB) grouped by the time (days) elapsed between the anti-TB treatment onset and the blood collection for this study (G.IV: 0–2 days; G.V: 3–6 days; and G.VI: ≥7 days).

Remarkably, in single gene analysis *NPC2* mRNA expression levels showed the highest discrimination, detecting 86% (25/29) of the TB (G.IV) patients and only one control (1/12), corresponding to 92% specificity (**Table 2**). Therefore, using *NPC2*^high^ expression for screening, in conjunction with the available clinical/radiological findings, allowed to differentiate latent and active forms of infections: G.I = 0/12 (0%), G.II = 0/24 (0%), G.III = 2/48 (4%), G.IV = 25/29 (86%), G.V = 8/12 (67%), G.VI = 1/4 (25%) (**Figure 3**). The use of combinations of the three mRNAs and/or other available epidemiological data did not improve classifying capability any further.

**Table 2:** Receiver operating characteristic (ROC) analysis of normalized expression values, via quantitative real-time PCR, to classify recent close TB contacts from G.I (low probability of latent tuberculosis infection) and patients with tuberculosis (G.IV) in the Brazilian cohort.

Gene	AUC	Sensitivity (%)	Specificity (%)	Cut-off point^¥^
		
	[95% CI]			
*DOCK9*	0.66 [0.46–0.86]	19 [7–39]	91.7 [62–100]	>0.481
*EPHA4*	0.73 [0.56–0.90]	53.4 [33–73]	91.7 [62–100]	>0.160
*NPC2*	0.88 [0.77–0.99]	84.6 [65–96]	91.7 [62–100]	>0.133


*^¥^normalized expression values.*

### RT-qPCR: Follow up Analysis

Among all rCt showing an *NPC2*^high^ expression profile (*NPC2*^high^/total: G.I = 1/12 [8.3%], G.II = 13/24 [54.2%], and G.III = 29/48 [60.4%]; total = 43/84 [51%]), the majority accepted the prophylactic treatment for LTBI (tLTBI/*NPC2*^high^: G.I = 0/1 [0%]; G.II = 4/13 [31%]; G.III = 25/29 [86%]; total = 29/43 [67%]). Of note, among all untreated rCt (*n* = 41), a single subject in G.III progressed to active TB during follow up (**Supplementary Table S3**). The mRNA expression profile of this participant was high only for *NPC2* at T_0_ (*DOCK9*: 0.070, *EPHA4*: 0.121 and *NPC2*: 0.248), which was even higher upon diagnosis of TB (*DOCK9*: 0.076, *EPHA4*: 0.247 and *NPC2*: 0.309) and decreased after the anti-TB treatment (*DOCK9*: 0.019, *EPHA4*: 0.065 and *NPC2*: 0.152) (**Supplementary Figure S2**). None of the *NPC2*^low^ participants progressed to active TB during the period of this study.

### Microarray Reanalysis: Cross-Sectional Studies

**FIGURE 4:**
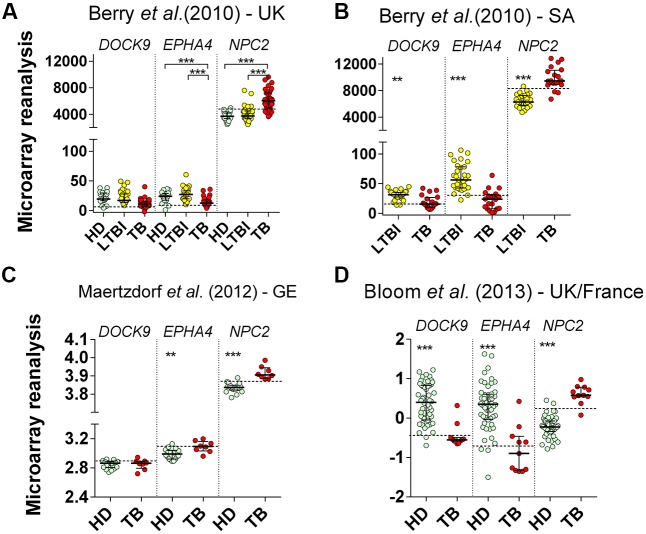
**Reanalysis of *DOCK9, EPHA4*, and *NPC2* expression in whole blood from healthy donors (HD), latently infected subjects (LTBI) and patients with pulmonary tuberculosis (TB) based on the data published by Berry et al. (2010), Maertzdorf et al. (2012), and Bloom et al. (2013).** The previous microarrays studies were performed in cohorts from: **(A)** UK (HD = 24, LTBI = 38, TB = 24), **(B)** South Africa (LTBI = 31, TB = 20), **(C)** Germany (HD = 18, TB = 8), and **(D)** UK/France (HD = 52, TB = 11), respectively. Mann–Whitney *U* or Kruskal–Wallis tests were performed for two or >2 groups comparisons, respectively (^∗∗^*p* ≤ 0.005 and ^∗∗∗^*p* < 0.001). Small lines, dotted lines and bars represent the median, cut-off and ±interquartile range, respectively.

All reanalyzed cohorts showed significant (*p* < 0.001) differential expression of *EPHA4* and *NPC2* mRNAs in whole blood of TB patients (**Figures 4A–D**). Specificities were high (≥94%) for all selected genes. In ROC curve analysis, again, the highest AUROC values (≥0.95) were observed for *NPC2* mRNA, corresponding to sensitivities between 82% (95% CI 66%–93%) and 100% (95% CI 63%–100%), followed by *EPHA4*, AUROC from 0.79% (95% CI 0.66%–0.91%) to 0.92% (95% CI 0.85%–1.0%), with poor-to-moderate sensitivities: 23.5% (95% CI 11%–41%) to 75% (95% CI 51%–91%) (**Table 3**). On the other hand, *DOCK9* mRNA, AUROC from 0.53% (95% CI 0.28%–0.82%) to 0.93% (95% CI 0.84%–1.0%), usually showed low sensitivities, from 13% (95% CI 3%–57%) to 35% (95% CI 15%–59%), except for the Bloom et al. (Bloom et al., 2013) study (82%, 95% CI 48%–98%) (**Table 3**).

**Table 3:** Receiver operating characteristic curve reanalysis of deposited microarray expression data for *DOCK9. EPHA4*, and *NPC2*, to classify healthy donors (HD) and tuberculosis (TB) cases in cohorts from the UK, South Africa, Germany, and France.

		Study site (reference)							
		
		UK^†^ (2)		South Africa^¥^ (2)		Germany (5)		UK/France (6)	
					
Gene		*n*	Value [95% CI]	*n*	Value [95% CI]	*n*	Value [95% CI]	*n*	Value [95% CI]
*DOCK9*	AUC (HD vs. TB)		0.79 [0.66–0.92]		0.80 [0.66–0.95]		0.53 [0.28–0.82]		0.93 [0.84–1.0]
	Specificity (%)	24	96 [79–100]	31	97 [83–100]	18	94 [73–100]	52	96. [87–100]
	Sensitivity (%)	34	15 [5–31]	20	35 [15–59]	8	13 [0.3–53]	11	82 [48–98]
*EPHA4*	AUC (HD vs. TB)		0.79 [0.66–0.91]		0.92 [0.85–1.0]		0.85 [0.69–1.0]		0.90 [0.79–1.0]
	Specificity (%)	24	96 [79–100]	31	97 [83–100]	18	94 [73–100]	52	962 [87–100]
	Sensitivity (%)	34	24 [11–41]	20	75 [51–91]	8	63 [25–92]	11	55[23–83]
*NPC2*	AUC (HD vs. TB)		0.95 [0.91–1.0]		0.97 [0.93–1.0]		0.99 [0.95–1.0]		0.99 [0.97–1.0]
	Specificity (%)	24	96 [79–100]	31	97 [83–100]	18	94 [73–100]	52	96 [87–100]
	Sensitivity (%)	34	82 [66–93]	20	85 [66–97]	8	100 [63–100]	11	91 [58–100]


*^†^Study population composed of immigrants from endemic countries or household contacts.*

*^¥^HD samples were not available; therefore, ROC analysis was performed comparing latently infected subjects and TB patients.*

### Microarray Reanalysis: TB Treatment follow up

**FIGURE 5:**
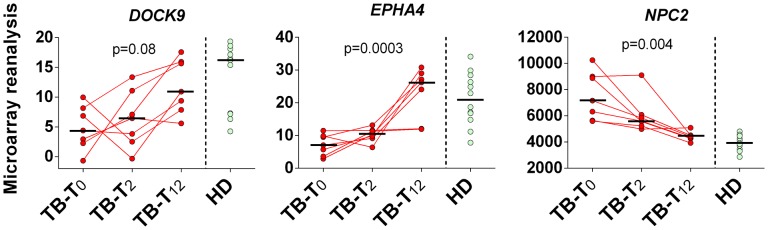
**Twelve months follow up of *DOCK9, EPHA4*, and *NPC2* expression after initiation of anti-TB treatment.** Pulmonary tuberculosis (TB, *n* = 7) was confirmed by isolation of *M. tuberculosis* upon culture of sputum or bronchoalvelolar lavage fluid. Samples from healthy donors (HD, *n* = 12) without known exposure to TB who were negative by both tuberculin skin test and interferon-gamma release assay were also included in the analysis. Whole blood samples from TB patients were collected prior to the initiation of the anti-mycobacterial therapy (TB-T_0_), in the second (TB-T_2_) and twelfth (TB-T_12_) consecutive months. Short bars represent medians. Reanalyzed data from Berry et al. (2010).

Reanalyzing microarray blood expression data from TB patients (*n* = 7) at enrollment (T_0_), and at T_2_ and T_12_ (elapsed time in months) after initiation of antimycobacterial treatment, we noticed a gradual trend of up-regulation of *DOCK9* (*p* = 0.08) and *EPHA4* mRNA (*p* = 0.0003), and down-regulation of *NPC2* (*p* = 0.004), during follow up (**Figure 5**). Even though *DOCK9* and *EPHA4* showed bimodal gene expression profiles among HD (*n* = 12) and TB-T_12_ groups, no statistical differences were observed between these groups for all target genes (Mann–Whitney *U*-test, *p* ≥ 0.415) and, notably, the *NPC2*^high^ biomarker was the only one that normalized completely 12 months after the onset of anti-TB treatment.

## Discussion

Previous whole blood genomic-scale studies pointed to a 144 (Bloom et al., 2013) or 393 (Berry et al., 2010) gene-transcript signature (including *DOCK9* and *EPHA4*) for TB, showing 61.7–94.1% sensitivities and 93.8–97% specificities (Berry et al., 2010; Bloom et al., 2013). Nevertheless, the use of 100s of genes for transcriptional profiling in clinical practice is currently impractical. Lu et al. (2011) proposed a smaller combination of genes, *CXCL10. ATP10A* and *TLR6*, which showed 71% sensitivity and 89% specificity to discriminate between latent and active TB. Lee et al. (2016) with a similar objective showed excellent AUROC ( = 1.0) for the comparisons between LTBI (*n* = 7) or active TB (*n* = 7) vs. HD (*n* = 7) using peripheral blood *ASUN* or *PTPRC* mRNA levels. Even though this accuracy was independently validated (AUROC ≥ 0.94), the assayed cohort had a small sample size (15 TB, 17 LTBI, and 15 HD; Lee et al., 2016). In a separate study, gene expression differentiation between TB and other pulmonary diseases was also described, which was based on another set of three genes (*GBP5. GZMA*, and *CD64*) and had a 93% sensitivity and 95% specificity; Laux da Costa et al., 2015). More recently, Zak et al. (2016), in a prospective African contact tracing study that featured a study design similar to ours (RNAseq for target-gene screening and RT-qPCR for biomarker validation), proposed a 16 gene-based transcriptional biosignature for risk of disease progression with 80.6% specificity, but with low sensitivity (66.1%) in the 12 months preceding TB diagnosis.

In the present study, we describe, for the first time, an up-regulation of *NPC2* mRNA levels in the blood of tuberculous subjects, as well as the transcription profiles of *DOCK9. EPHA4* and *NPC2* among Brazilians recently exposed to a TB index case. As 25% (1/4) of the untreated subjects from G.III progressed to active TB during the 1-year follow up period (**Supplementary Table S3**), the prophylactic treatment might have compromised the evaluation of the proposed biomarker in the early detection of TB. Another limitation of this study is the lack of an unexposed control group among the Brazilian donors. However, in high TB-burden scenarios, as Rio de Janeiro/Brazil, it is difficult to rule out a previous exposure to *M. tuberculosis*, even in subjects with no reported contact with a TB index case. To overcome this, we opted to group the enrolled participants according to baseline and follow up response to TST, a highly sensitive LTBI test (Schluger and Burzynski, 2010), and evaluated HD expression profiles in other cohorts. Notably, a one-gene biosignature, *NPC2*^high^, showed high sensitivity (85%) and specificity (92%), via RT-qPCR, for TB detection. In addition, a reanalysis of expression data from public repositories showed a similar sensitivity (82–100%) and specificity (94–97%) in populations with different genetic backgrounds (Berry et al., 2010; Maertzdorf et al., 2012; Bloom et al., 2013).

Here, the monitoring of *NPC2* mRNA levels in blood could be used as a screening tool for the detection of active TB. On the other hand, by RT-qPCR or microarray reanalysis, *DOCK9* (13–82%) and *EPHA4* (24–75%) usually showed poor-to-moderate sensitivities but, interestingly, they exhibited a dichotomous median expression profile among the different TB groups in comparison to the respective control: down-modulation in populations from the UK and South Africa (**Figure 4**), but the contrary among Brazilians (**Figures 2B,C**) and Germans (**Figure 4C**). A bimodal *DOCK9* and *EPHA4* mRNA expression profile was also observed, principally in the TB groups from Brazil (G.IV and G.V, **Figures 2B,C**), UK cross-sectional (**Figure 4D**) and the follow up cohorts (**Figure 4**). This bimodal transcription, in response to physiological disturbances, was previously described for other genes (Bessarabova et al., 2010). Furthermore, according to Bessarabova et al. (2010) the presence of a bimodal gene expression profile could correspond to different disease subtypes. Thus, further studies are needed to assess whether different *M. tuberculosis* strains, infection sites, previous infections, host genetic background or co-morbidities can affect *DOCK9* and *EPHA4* mRNA expression profiles. Interestingly, this bimodal behavior was not observed for *NPC2*.

Anti-TB chemotherapy usually requires a 6-months standard regimen to eradicate the infection (Jindani et al., 2004). The observed change trend in the median expression levels for all target genes toward an uninfected control-like transcriptional profile, in the beginning of treatment (G.VI, **Figure 2**), and more pronounced in the TB-T_12_ group (**Figure 3**), is an indication that these modulations are dependent on the presence of viable bacilli in the host system.

The exact physiological role of the dedicator of cytokinesis 9 (Dock9) protein has not been totally defined yet. According to Gadea and Blangy (2014), it is possible that Dock9 plays a role in the activation of Cdc42, a Rho GTPase that can affect TGF-beta1-mediated transcriptional responses. Dock9 has also been implicated in the epithelial to mesenchymal cell transition through the reorganization of the actin cytoskeleton and the regulation of cell polarity (Brown et al., 2008; Gadea and Blangy, 2014). Previous studies have pointed to the role of Epha4 and its receptor, Ephrin A1, in the process of lymphocyte and monocyte adhesion (Poitz et al., 2015). The Eph/Ephrin-system, among others, was described to influence Rho GTPases, such as Cdc42 (Singh et al., 2012). Taken together, these observations suggest that these proteins have a common pathway in the recruitment of immune cells to *M. tuberculosis* infection sites.

*NPC2* plays roles in cholesterol and glycolipid trafficking and/or transport, and mutations in it are causally related to Niemann-Pick disease, a life-threatening lysosomal storage disease (Park et al., 2003), but studies on its role in *M. tuberculosis* pathogenesis are scarce. Recently, knockdown experiments demonstrated that adding all-*trans* retinoic acid (ATRA), the biologically active form of vitamin A, to *M. tuberculosis*-infected human monocyte cultures resulted in an *NPC2*-dependent decrease in total cellular cholesterol, associated with a consequent increase in antimicrobial activity through lysosomal acidification (Wheelwright et al., 2014). The authors postulated that decreasing levels of *NPC2* expression might favor *M. tuberculosis* persistence. In contrast, we observed an up-regulation of *NPC2* mRNA in whole blood from patients with active TB and LTBI subjects, indicating that increased *NPC2* expression is part of the host response to TB infection but does not confer protection in all cases. As we could observe in the incident case (**Supplementary Figure S2**), and in the subcohort of *NPC2*^high^ TB patients in whom disease progression could not be prevented, expression of this gene was still high. Our data indirectly demonstrate that upon the persistence of *M. tuberculosis*, systemic modulation of gene expression may occur even in asymptomatic, but latently infected subjects. Nonetheless, it is still necessary to investigate which genotypic or phenotypic features could be responsible for the *NPC2*^low^ expression profile in a few TB patients. Moreover, it is also necessary to clarify whether NPC2 up-regulation is specific for TB infection among patients with respiratory symptoms. In two of the reanalyzed studies (Maertzdorf et al., 2012; Bloom et al., 2013) samples from other pulmonary diseases were also included (data not shown). The *NPC2*^high^ blood expression profile was detected in only 1/6 (17%) pneumonia patients and 1/8 (13%) lung cancer patients (Bloom et al., 2013) but, in 50% (*n* = 9/18) (Maertzdorf et al., 2012) and 56% (*n* = 9/16) (Bloom et al., 2013) of patients with active sarcoidosis. As sarcoidosis is a low incidence disease (Nunes et al., 2007), of which *M. tuberculosis* is a possible aetiological agent (van Enschot and van Balkom, 2013), the observed relatively frequent detection of the *NPC2*^high^ biomarker should not compromise the application of this marker for the screening of active TB cases.

Considering the limitations of available routine methods for screening of *M. tuberculosis* infection among respiratory symptomatic or asymptomatic individuals, which hamper the control of this important illness, the profiling of *NPC2* mRNA expression in blood seems to be an attractive alternative, since it has demonstrated an excellent accuracy using a fast methodology such as RT-qPCR for single-gene quantification in blood specimens. The additional similar results, obtained with populations of various genetic backgrounds and from different TB-burden scenarios, encourage us to stress the need for further validations of this biomarker in new cohorts, evaluating its modulation during disease progression and treatment success. In the meantime, the roles of *DOCK9. EPHA4*, and *NPC2* genes in TB pathogenesis also merit further investigations.

## Author Contributions

LA, FP, and MS designed the study. LA and MS wrote the manuscript. LV, MR-A, FM, AA, AM, AK, JL, MM, and FP modified the manuscript. Human samples were obtained by LA, MR-A, FM, AA, AM, AK, JL, MM, and MS. LA and AA performed the RT-qPCR experiments. LA and MR-A performed the microarray reanalysis. LA, LV, and MR-A conducted statistical analysis. All authors reviewed the results, made substantial contributions and approved the final version of the manuscript.

## Conflict of Interest Statement

The authors declare that the research was conducted in the absence of any commercial or financial relationships that could be construed as a potential conflict of interest.
