# Supplementary material for: Transcriptomic Biomarkers for Tuberculosis: Evaluation of DOCK9. EPHA4, and NPC2 mRNA Expression in Peripheral Blood
Source: Front Microbiol. 2016 Oct 25;7:1586. doi: 10.3389/fmicb.2016.01586 (PMC5078140; doi:10.3389/fmicb.2016.01586)
Supplement: Supplementary file 1 [file Table_1.DOCX]

Supplementary Material

**Host RNA biomarkers for tuberculosis: evaluation of *DOCK9, EPHA4*, and *NPC2* expression modulations in blood.**

Leonardo Silva de Araujo, Lea A. I. Vaas, Marcelo Ribeiro-Alves, Fernanda Carvalho Queiroz Mello , Alexandre Silva de Almeida, Adriana da Silva Resende Moreira, Afrânio Lineu Kritski, José Roberto Lapa e Silva, Milton Ozório Moraes, Frank Pessler, and Maria Helena Féres Saad.

**Corresponding author:** Dr. Maria Helena Féres Saad: [saad@ioc.fiocruz.br](mailto:saad@ioc.fiocruz.br)

**Supplementary Table S1 -** Forward and reverse primers used for the RT-qPCR amplifications.

| Sense | Gene | Sequence |
| --- | --- | --- |
| Forward | *DOCK9* | 5’-AGAGCCACACAGGAAGAAGTC-3’ |
|  | *EPHA4* | 5’-ATGCAGTGGGCGTCTCAAAG-3’ |
|  | *NPC2* | 5’- GTCCCAGTTCCCTTTCCCAT-3’ |
|  | *RPL13A* | 5’-GACAAGAAAAAGCGGATGGT-3’ |
| Reverse | *DOCK9* | 5’-TGGCTCTCCAATCCTTCCTCA-3’ |
|  | *EPHA4* | 5’-TGGTTTACATTTAGTGACCACGCC-3’ |
|  | *NPC2* | 5’-ACTCCACCACCAGTTTTATAGAGG-3’ |
|  | *RPL13A* | 5’-GTACTTCCAGCCAACCTCGT-3’ |
