# Supplementary material for: Transcriptomic Biomarkers for Tuberculosis: Evaluation of DOCK9. EPHA4, and NPC2 mRNA Expression in Peripheral Blood
Source: Front Microbiol. 2016 Oct 25;7:1586. doi: 10.3389/fmicb.2016.01586 (PMC5078140; doi:10.3389/fmicb.2016.01586)
Supplement: Supplementary file 2 [file Table_2.DOCX]

Supplementary Material

**Host RNA biomarkers for tuberculosis: evaluation of *DOCK9, EPHA4*, and *NPC2* expression modulations in blood.**

Leonardo Silva de Araujo, Lea A. I. Vaas, Marcelo Ribeiro-Alves, Fernanda Carvalho Queiroz Mello , Alexandre Silva de Almeida, Adriana da Silva Resende Moreira, Afrânio Lineu Kritski, José Roberto Lapa e Silva, Milton Ozório Moraes, Frank Pessler, and Maria Helena Féres Saad.

**Corresponding author:** Dr. Maria Helena Féres Saad: [saad@ioc.fiocruz.br](mailto:saad@ioc.fiocruz.br)

**Supplementary Table S2 -** Mean and median normalized expression values of *DOCK9*, *EPHA4*, and *NPC2* mRNAs in the whole blood from the Brazilian cohort.

|  |  | DOCK9 | | EPHA4 | | NPC2 | |
| --- | --- | --- | --- | --- | --- | --- | --- |
|  | n | Mean | Median | Mean | Median | Mean | Median |
| G.I | 12 | 0.35273 | 0.02034 | 0.10958 | 0.08196 | 0.07996 | 0.07068 |
| G.II | 24 | 0.17688 | 0.0388 | 1.1611 | 0.12985 | 0.84109 | 0.14551 |
| G.III | 48 | 0.0955 | 0.03375 | 0.43779 | 0.13999 | 0.33744 | 0.15117 |
| G.IV | 29 | 0.26413 | 0.04446 | 0.98352 | 0.21672 | 1.18807 | 0.38236 |
| G.V | 12 | 2.07209 | 0.07288 | 2.31874 | 0.17185 | 1.93403 | 0.20722 |
| G.VI | 4 | 0.02995 | 0.01725 | 0.11665 | 0.10416 | 0.16408 | 0.16443 |
