# Supplementary material for: Transcriptomic Biomarkers for Tuberculosis: Evaluation of DOCK9. EPHA4, and NPC2 mRNA Expression in Peripheral Blood
Source: Front Microbiol. 2016 Oct 25;7:1586. doi: 10.3389/fmicb.2016.01586 (PMC5078140; doi:10.3389/fmicb.2016.01586)
Supplement: Supplementary file 3 [file Table_3.DOCX]

Supplementary Material

**Host RNA biomarkers for tuberculosis: evaluation of *DOCK9, EPHA4*, and *NPC2* expression modulations in blood.**

Leonardo Silva de Araujo, Lea A. I. Vaas, Marcelo Ribeiro-Alves, Fernanda Carvalho Queiroz Mello , Alexandre Silva de Almeida, Adriana da Silva Resende Moreira, Afrânio Lineu Kritski, José Roberto Lapa e Silva, Milton Ozório Moraes, Frank Pessler, and Maria Helena Féres Saad.

**Corresponding author:** Dr. Maria Helena Féres Saad: [saad@ioc.fiocruz.br](mailto:saad@ioc.fiocruz.br)

**Supplementary Table S3 -** Clinical and pharmacological outcome (medical release, treatment for latent tuberculosis infection [tLTBI] or for active pulmonary tuberculosis [TB]) according to the *M. tuberculosis* infected-like patterns of gene expression (NPC2^high^) at whole blood of healthy recent close contacts (rCt), stratified in 3 groups according to the probability of latent tuberculosis infection: (G.I) very low, (G.II) low to moderate and (G.III) moderate to high; and TB patients grouped by the time (days) relapsed between the anti-tuberculosis treatment onset and the blood collection for this study (G.IV: 0-2 days; G.V: 3-6 days and G.VI: >7 days).

| **Groups**  **(n)** | ***NPC2*^low^** | | | | ***NPC2*^high^** | | | |
| --- | --- | --- | --- | --- | --- | --- | --- | --- |
|  | Clinical outcome | | | | Clinical outcome | | | |
|  | n | Medical Release or  Refused tLTBI | tLTBI | Treatment for TB | n | Medical Release or  Refused tLTBI | tLTBI | Treatment for TB |
| G.I  (12) | 11 | 11 | - | - | 1 | 1 | - | - |
| G.II  (24) | 11 | 9 | 2 | - | 13 | 9 | 4 | - |
| G.III  (48) | 19 | 7 | 12 | - | 29 | 4^¥^ | 25 | - |
| G.IV  (29) | 4 | - | - | 4 | 25 | - | - | 25 |
| G.V  (12) | 4 | - | - | 4 | 8 | - | - | 8 |
| G.VI  (4) | 1 | - | - | 1 | 3 | - | - | 3 |
| **Total**  (129) | 53 | 29 | 15 | 9 | 76 | 12 | 29 | 36 |

¥ In this group 1/4 (25%) individual was clinically diagnosed with LTBI, also showing *M. tuberculosis* infected-like patterns of gene expression (*NPC2* >0.133), refused the prophylactic tLTBI, in approximately 4 months follow up this subject developed pulmonary TB.
