# Supplementary material for: Transcriptomic Biomarkers for Tuberculosis: Evaluation of DOCK9. EPHA4, and NPC2 mRNA Expression in Peripheral Blood
Source: Front Microbiol. 2016 Oct 25;7:1586. doi: 10.3389/fmicb.2016.01586 (PMC5078140; doi:10.3389/fmicb.2016.01586)

Supplementary Material

Host RNA biomarkers for tuberculosis: evaluation of *DOCK9*, *EPHA4*, and *NPC2* expression modulations in blood.

Leonardo Silva de Araujo, Lea A. I. Vaas, Marcelo Ribeiro-Alves, Fernanda Carvalho Queiroz Mello, Alexandre Silva de Almeida, Adriana da Silva Resende Moreira, Afrânio Lineu Kritski, José Roberto Lapa e Silva, Milton Ozório Moraes, Frank Pessler, and Maria Helena Féres Saad.

Corresponding author: Dr. Maria Helena Féres Saad: saad@ioc.fiocruz.br;

Dr Frank Pessler, MD: Pessler.Frank@mh-hannover.de.

Supplementary Figure 1 - ROC curves and the respective area under the curve (AUC) with 95% confidence interval (blue) comparing G.I (very low probability of LTBI) versus G.III (high probability of LTBI) and/or G.IV (tuberculosis).

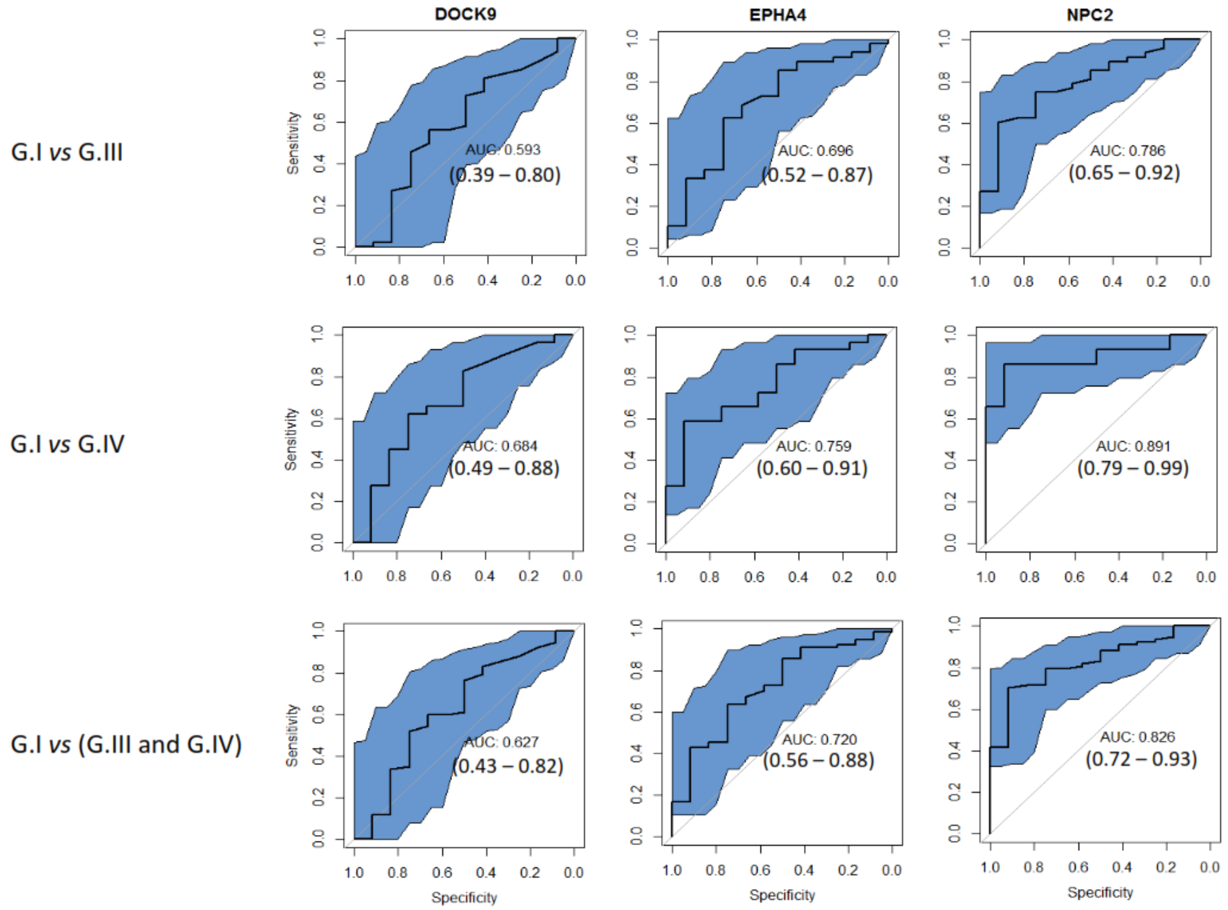

Supplement: Supplementary file 4 [file Image_1.PDF]
