# Supplementary material for: Transcriptomic Biomarkers for Tuberculosis: Evaluation of DOCK9. EPHA4, and NPC2 mRNA Expression in Peripheral Blood
Source: Front Microbiol. 2016 Oct 25;7:1586. doi: 10.3389/fmicb.2016.01586 (PMC5078140; doi:10.3389/fmicb.2016.01586)

Supplementary Material

Host RNA biomarkers for tuberculosis: evaluation of *DOCK9*, *EPHA4*, and *NPC2* expression modulations in blood.

Leonardo Silva de Araujo, Lea A. I. Vaas, Marcelo Ribeiro-Alves, Fernanda Carvalho Queiroz Mello , Alexandre Silva de Almeida, Adriana da Silva Resende Moreira, Afrânio Lineu Kritski, José Roberto Lapa e Silva, Milton Ozório Moraes, Frank Pessler, and Maria Helena Féres Saad.

Corresponding author: Dr. Maria Helena Féres Saad: saad@ioc.fiocruz.br;

Dr Frank Pessler, MD: Pessler.Frank@mh-hannover.de.

Supplementary Figure S2 - *DOCK9*, *EPHA4* and *NPC2* follow up normalized expression profiling, via real time quantitative PCR, of a single tuberculosis (TB) progressor; at enrolment with latent TB (LTBI, G.III), upon diagnoses of TB (G.IV) and after $\geq$7 days of anti-TB treatment onset (G.VI).

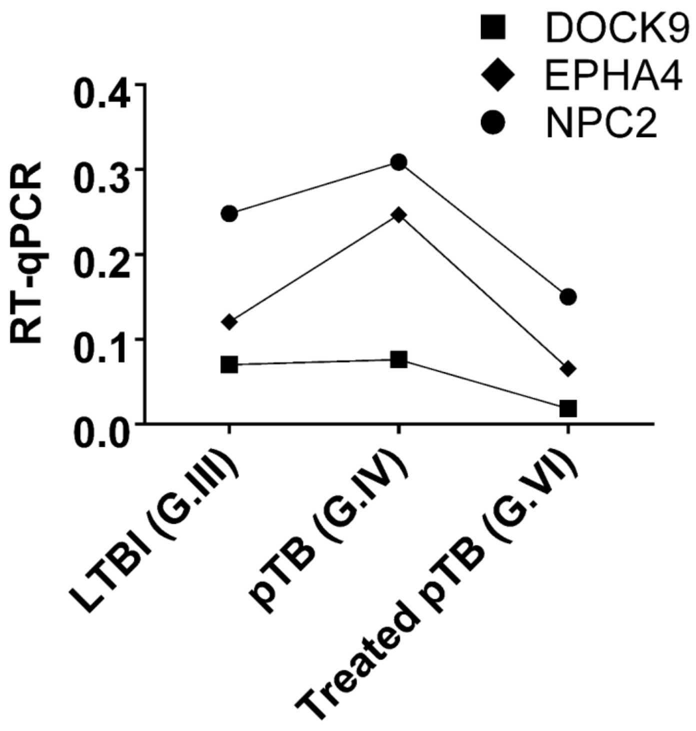

Supplement: Supplementary file 5 [file Image_2.PDF]
